# Knowledge as a Tool Against Stigma: How Mental Health Literacy Influences Attitudes and Behaviors Among Medical Students

**DOI:** 10.1192/j.eurpsy.2026.11828

**Published:** 2026-07-31

**Authors:** G. Chakchouk, N. Regaieg, Y. Trabelsi, F. Guermazi, I. Feki, J. Masmoudi, I. Baati

**Affiliations:** Department of Psychiatry A, Hedi Chaker Hospital, Sfax, Tunisia

## Abstract

**Introduction:**

Several studies have explored mental health stigma using the Knowledge–Attitudes–Behaviors (KAB) model. This model highlights the major role of mental health literacy in shaping stigmatizing attitudes and behaviors.

**Objectives:**

This study aims to: measure the level of mental health literacy in a sample of medical students determine demographic and experiential factors associated with this knowledge examine how mental health knowledge relates to other dimensions of stigma

**Methods:**

A cross-sectional study was conducted among medical students enrolled at the faculty of medicine of Sfax, Tunisia, from February to October 2025. Participants completed via a Google form survey a questionnaire including sociodemographic characteristics and personal experiences with mental health.

Psychometric evaluation was performed using the: French version of the Community attitudes towards the mentally ill (CAMI) scale, the Mental Health Knowledge Schedule (MAKS) and the Reported and intended behavior scale (RIBS).

**Results:**

The mean MAKS score was 32.5 ± 4.3. The mean RIBS score was 13.4 ± 3.1. The overall CAMI mean score was 15.2 ± 3.0, with subscales’ means of 14.0 ± 3.3 for Authoritarianism, 27.8 ± 5.2 for Benevolence, 19.3 ± 4.8 for Social Restrictiveness, and 16.7 ± 2.8 for Community Ideology.

Students who had previously consulted a mental health professional showed higher MAKS scores compared to those who had not (p = 0.042). MAKS scores differed significantly across years of medical training (p = 0.001). Fourth-year students showed the highest scores (mean rank = 53.2).

The analyses revealed that higher mental health knowledge was significantly associated with lower levels of stigmatizing attitudes: MAKS scores were negatively correlated with the total CAMI score (p <0.001) and with the stigma-related CAMI dimensions: Authoritarianism (p <0.001) and Social Restrictiveness (p <0.001).

Furthermore, MAKS scores were positively correlated with Benevolence (p <0.001) and Community Ideology (p <0.001), indicating that greater mental health knowledge has an impact on all aspects of attitudes regarding mental illness. Positive correlations were also observed between MAKS and RIBS (p <0.001) indicating that the impact of greater knowledge about mental illness not only on attitudes but also on intended behaviors towards the mentally ill.

**Conclusions:**

Our findings provide evidence that greater mental health knowledge is associated with more inclusive, empathetic, and less stigmatizing attitudes toward mental illness.

**Disclosure of Interest:**

None Declared

